# Management of Complex Facial Lacerations in the Emergency Department

**DOI:** 10.5811/cpcem.2017.2.33270

**Published:** 2017-05-09

**Authors:** Austin Badeau, Shadi Lahham, Megan Osborn

**Affiliations:** * University of California, Irvine, Department of Plastic Surgery, Orange, California; † University of California, Irvine, Department of Emergency Medicine, Orange, California

## Abstract

Laceration injuries comprise over 8% of all emergency department (ED) visits annually.^1^ Given that laceration injuries represent a significant volume of ED visits, emergency physicians (EP) should be comfortable treating these types of injuries. We present the case of a 34-year-old male who presented to the ED as a trauma activation who suffered multiple injuries including complex full-thickness lacerations to his face. While there are scenarios in which consulting a specialist is necessary, knowledge and application of basic wound closure principles allows for many complex lacerations to be repaired by EPs. We provide a helpful systematic approach to evaluating and treating complex facial lacerations in the ED.

## INTRODUCTION

Approximately eight million patients present to emergency departments (ED) in the United States every year with laceration injuries.^1^ These numbers do not reflect lacerations seen and repaired in urgent care centers. Given that laceration injuries represent a significant volume of ED visits, emergency physicians (EP) should be comfortable treating these injuries. We present a patient who suffered a complex facial laceration that was managed in the ED.

## CASE REPORT

**Image 1:**
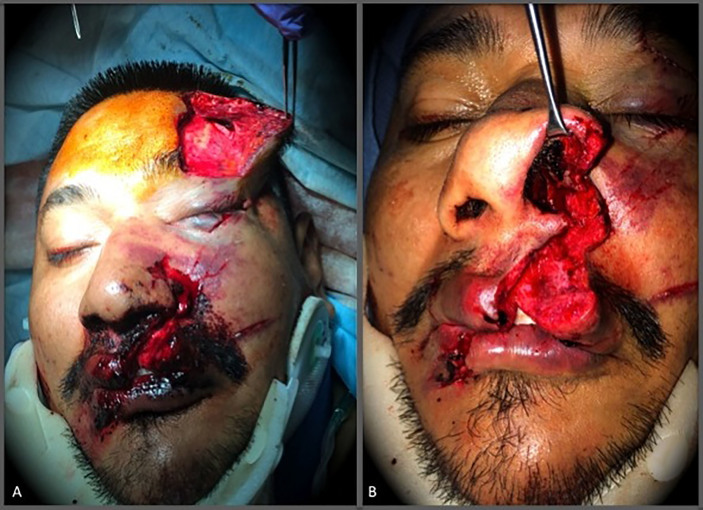
A) Full-thickness left forehead laceration. B) Full-thickness upper lip laceration extending through the floor of the nose and nasal ala.

A 34-year-old male presented to the ED as a trauma activation. He was the restrained driver in a single-car motor vehicle accident in which he sustained burns to the bilateral upper extremities, left-sided rib fractures, a right femoral neck fracture, open right tibia/fibula fractures, and full-thickness lacerations to his face. There were two main facial lacerations. The first was a stellate left forehead laceration that extended deep through the frontalis muscle and inferiorly through the left eyebrow (Image 1A). The second was a through-and-through upper lip laceration that extended into and violated the floor of the nose (Image 1B).

**Image 2:**
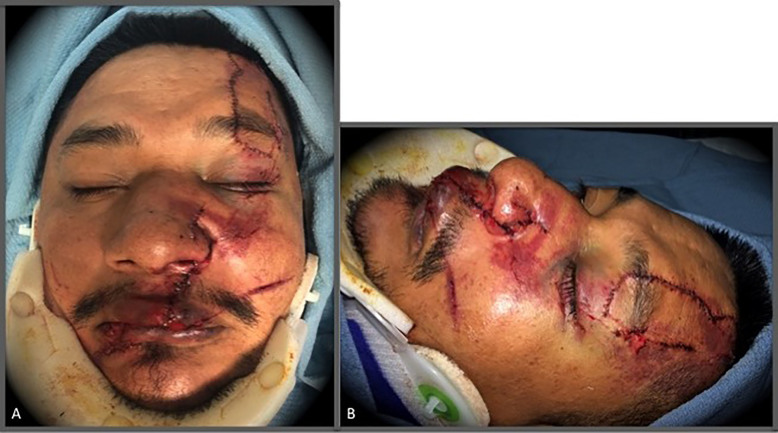
A) Repaired left forehead, upper lip, and nasal lacerations. B) Repaired left forehead, upper lip, and nasal lacerations.

Laceration repair began with providing anesthesia via a combination of local infiltration and targeted nerve blocks. The lacerations were then copiously irrigated with sterile saline. The forehead laceration was closed in a layered fashion using 4-0 Monocryl to re-approximate the frontalis muscle; buried interrupted 4-0 Monocryl placed in the deep dermis; and, lastly, 5-0 fast absorbing gut suture placed in a simple interrupted manner to close the epidermis (Image 2A). The upper lip laceration was closed in a similar layered fashion using 4-0 Monocryl to re-approximate the orbicularis oris muscle; buried interrupted 4-0 Monocryl placed in the deep dermis; and 5-0 fast absorbing gut suture placed in a simple interrupted manner to close the epidermis and dry vermillion of the lip (Image 2A/B). The internal oral and nasal mucosa was closed with 4-0 Vicryl suture in a simple interrupted manner.

## DISCUSSION

Laceration injuries comprise over 8% of all ED visits annually with up to 28% of these lacerations involving the face.^1^ Simple, superficial lacerations are often repaired by EPs, while more complex lacerations in cosmetically and/or functionally sensitive regions are often deferred to plastic surgeons, otolaryngologists, and oral-maxillofacial surgeons. This is especially true at tertiary-care academic medical centers where a full armamentarium of surgical specialists is readily available. While there are scenarios in which consulting a specialist is necessary, knowledge and application of basic wound closure principles allows for many complex lacerations to be repaired by EPs.

Initial evaluation of all traumatic facial lacerations should begin after providing adequate anesthesia. This can be achieved through a combination of short- and long-acting local anesthetics such as lidocaine and bupivicaine respectively. Anesthetics containing epinephrine will help decrease bleeding in the operative field and decrease the systemic distribution and potential toxicity of the local anesthetic. Additionally, 1ml of bicarbonate per 10ml of local anesthetic can also be added to the anesthetic mixture to neutralize the acidity of the anesthetic solution, increase the duration of action of the anesthetic, and decreases the pain with injection. Nearly painless local anesthesia can be provided by using a bicarbonate buffering solution, injecting with a small (27–30G) needle, and by always keeping a wheel of local anesthetic ahead of the needle while injecting.^2^ One should consider using targeted nerve blocks when possible for multiple lacerations in the same neurosensory distribution.^3^ This method can provide adequate anesthesia and decreases the total dosage of anesthesia. Nerve blocks also offer the advantage of providing anesthesia more remotely without distorting the local anatomy of the tissue needing repair, as opposed to when anesthetic is injected locally.^3^ This issue is especially important when attempting to re-approximate linear and cosmetically sensitive areas such as the lips, eyebrows, ears, etc.^3^

Tap water or saline should then be used to copiously irrigate the wound. Thorough irrigation removes all macroscopic debris, helps identify active bleeding, and allows for adequate visual exploration of the wound. During inspection of the wound, active venous or arterial bleeding should be addressed with cautery or sutures. Adequate hemostasis is especially important when closing avulsion flaps, which can develop large hematomas. Visual and manual exploration of the wound should look to identify the peripheral and deep extent of the wound. A hemostat can be used to probe for injuries communicating from one anatomic space to another. Conservative sharp debridement of ragged, severely contused, and devitalized tissue should also be performed.^4^ Lastly, visual inspection should aim to identify any major nerve, duct or other structural injuries that would require surgical consultation and operative repair.^4^

The basic principle of closing all lacerations is to realign anatomic structures (superficial and deep) in a tension-free manner. Performing a layered closure helps facilitate this goal by distributing tension into the deeper, strong soft-tissue layers so that the epidermis is nearly “kissing” by the time it is sutured closed. Muscle is generally the deepest layer that requires closure in the face. In the forehead and brow, the frontalis muscle and orbicularis oculi should be re-approximated using a 4-0 absorbable monofilament suture. In full-thickness lip lacerations the orbicularis oris muscle should similarly be brought together. Muscle tissue can be difficult to re-approximate, as the tissue is relatively weak causing sutures to tear through the fibers even under minimal tension; this is especially true in contused muscle. Distributing the tension of a suture over a greater surface area of muscle using a horizontal mattress suture can help mitigate this problem. Most importantly, it is important to identify and include the superficial fascia of the muscle during re-approximation because this strong fibrous component of the muscle will hold suture under normal tension. Failure to perform a proper muscle layer closure will result in non-contiguous healing of the muscle, which may cause animation deformities and depressed wide scars.

CPC-EM Capsule

What do we already know about this clinical entity?

Annually, over 8 million patients present to emergency departments in the US with laceration injuries. Emergency physicians should be comfortable treating the majority of these injuries.

What makes this presentation of disease reportable?

In our ever-specializing medical environment, this case demonstrates repair of complex facial lacerations repaired in the Emergency Department without sub-specialist consultation.

What is the major learning point?

An understanding of and adherence to basic traumatic wound care principles allows for the management of complex laceration repairs to be performed by Emergency physicians.

How might this improve emergency medicine practice?

For providers practicing in resource-limited environments, we demonstrate the type of laceration repairs that can be performed in the ED when surgical consultation is unavailable.

The next layer to be closed is the deep dermis and subcutaneous fat. This closure is best carried out with a 4-0 absorbable monofilament suture that is placed in simple interrupted manner with the knot buried. The suture needle should enter the subcutaneous fat and exit near the dermal-epidermal junction on one side of the laceration where it will then travel across the wound and enter the dermal-epidermal junction and exit the subcutaneous tissue of the opposing side. Precise alignment of this layer is important for scar healing.

The epidermis is the most superficial layer that requires closure. When repairing the lip, it is crucial to precisely align the vermillion border where even a 1mm discrepancy is discernable at conversational distance. This is best achieved with a 5-0 or 6-0 non-absorbable monofilament suture; a similar size un-dyed rapidly dissolving suture will also work and has the advantage of not needing to be removed. EPs should be especially mindful of the burden of suture removal in young children and patients with poor follow-up. When permanent sutures are placed in the epidermis of the face, they should be removed in 5–7 days to prevent the unsightly “train-track” appearance of a scar where sutures have been left in for too long. Consider using a brightly dyed non-absorbable suture when repairing lacerations extending into the scalp of patients with dark hair, as this will make suture removal much easier. In either suture choice, the laceration should be repaired in a simple interrupted manner. Longer lacerations can be repaired with a continuous “baseball” stitch to increase speed of the repair. This advantage should be weighed against the disadvantage of relying on a single continuous stitch for the epidermal repair where rupture of the suture or tearing of the skin compromises the entire length of the repair.

Tissue glue, such as Dermabond, can also be used to close the epidermal layer of skin. In very superficial lacerations that do not fully penetrate the dermis, Dermabond can be used alone to re-approximate the superficial dermis and epidermis. Using Dermabond in this way is especially useful in pediatric patients where suturing may require monitored sedation. Dermabond is most commonly used in conjunction with other deep and superficial suture layers. Sealing wounds with Dermabond in this fashion adds another layer of strength and provides a watertight closure, which can be washed and left open to the air without concern for contamination from physical debris and bacteria. Of note, previous studies are undecided on the use of antibiotics. In wounds with high risk for infection consider starting prophylactic antibiotics.^5^

Nasal and oral mucosa should be repaired in a simple interrupted manner with a soft, braided absorbable suture. When repairing lip lacerations the oral mucosa should be repaired after the muscle layer is re-approximated, but before closure of the epidermis.

Most lacerations will be treated in regular, perhaps dimly lit, rooms rather than trauma bays with adjustable overhead spotlights. Accordingly, a camping-style headlamp is an invaluable and relatively cheap tool that EPs should purchase and can use for a variety of procedures including laceration repair. Lastly, always try to use a flat-surfaced needle holder/driver while suturing, especially when using small needles. Most EDs stock or can acquire separate surgical grade flat-surfaced needle holder/drivers. Unfortunately, pre-packaged suture kits often contain needle holder/drivers with serrations through which small needles can rotate and slide. This can be especially frustrating and time consuming when repairing large lacerations.

Closing complex facial lacerations is a professionally rewarding procedure. While some lacerations may require surgical consultation, many complex lacerations can be treated immediately by EPs with arrangement for sub-specialist follow-up on cosmetically and/or functionally sensitive injuries. Time constraints are a significant obstacle that may preclude treating these types of complex facial injuries in the ED. This is especially true in single coverage EDs; conversely single coverage EDs may also be less likely to have on-call surgical sub-specialists available to treat these injuries. Management of large complex lacerations should be in the scope of all EPs and is a necessity for those whose practice includes rural emergency medicine, wilderness medicine, cruise ship medicine, expedition medicine, etc. When faced with any laceration injury, remember to first provide complete local anesthesia using local nerve blocks when possible. Next, perform thorough irrigation and exploration of the wound. Lastly, perform a multi-layered closure of the wound to achieve a tension-free re-alignment of the deep and superficial anatomy. Applying this approach should allow all EPs to successfully tackle a high percentage of laceration injuries.
